# Primary-Sjögren’s-Syndrome-Related Interstitial Lung Disease: A Clinical Review Discussing Current Controversies

**DOI:** 10.3390/jcm12103428

**Published:** 2023-05-12

**Authors:** Gaetano La Rocca, Francesco Ferro, Gianluca Sambataro, Elena Elefante, Silvia Fonzetti, Giovanni Fulvio, Inmaculada C. Navarro, Marta Mosca, Chiara Baldini

**Affiliations:** 1 Rheumatology Unit, Department of Clinical and Experimental Medicine, University of Pisa, Via Roma 67, 56126 Pisa, Italy; francescoferrodoc@gmail.com (F.F.); elena.elefante87@gmail.com (E.E.); silvia.fonzetti@gmail.com (S.F.); giovanni.fulvio92@gmail.com (G.F.); i.navarrogarcia@studenti.unipi.it (I.C.N.); marta.mosca@unipi.it (M.M.); chiara.baldini74@gmail.com (C.B.); 2 Department of Clinical and Experimental Medicine, Regional Referral Centre for Rare Lung Diseases, A.O.U. Policlinico “G. Rodolico-San Marco”, University of Catania, Via Santa Sofia 78, 95124 Catania, Italy; dottorsambataro@gmail.com; 3 Artroreuma S.R.L., Rheumatology Outpatient Clinic Associated with the National Health System, Corso S. Vito 53, 95030 Catania, Italy

**Keywords:** Sjögren’s, interstitial lung disease, lung ultrasound, patterns, prognosis, treatment

## Abstract

Lung involvement, especially interstitial lung disease, is a potentially severe extra-glandular manifestation of Primary Sjogren’s Syndrome (pSS-ILD). ILD can manifest either as a late complication of pSS or anticipate sicca symptoms, likely reflecting two different patho-physiological entities. Presence of lung involvement in pSS subjects can remain subclinical for a long time; therefore, patients should be actively screened, and lung ultrasound is currently being investigated as a potential low cost, radiation-free, easily repeatable screening tool for detection of ILD. In contrast, rheumatologic evaluation, serology testing, and minor salivary gland biopsy are crucial for the recognition of pSS in apparently idiopathic ILD patients. Whether the HRCT pattern influences prognosis and treatment response in pSS-ILD is not clear; a UIP pattern associated with a worse prognosis in some studies, but not in others. Many aspects of pSS-ILD, including its actual prevalence, association with specific clinical–serological characteristics, and prognosis, are still debated by the current literature, likely due to poor phenotypic stratification of patients in clinical studies. In the present review, we critically discuss these and other clinically relevant “hot topics” in pSS-ILD. More specifically, after a focused discussion, we compiled a list of questions regarding pSS-ILD that, in our opinion, are not easily answered by the available literature. We subsequently tried to formulate adequate answers on the basis of an extensive literature search and our clinical experience. At the same, we highlighted different issues that require further investigation.

## 1. Introduction

Primary Sjögren’s Syndrome (pSS) is an autoimmune exocrinopathy classically presenting with sicca symptoms (i.e., xeropthalmia and xerostomia) as a result of lacrimal and salivary gland lymphocytic infiltration. Nonetheless, pSS is a systemic autoimmune disorder leading to extra-glandular manifestations in 30–40% of affected patients [1].

The EULAR Sjögren’s Syndrome disease activity index (ESSDAI) includes, in the pulmonary domain, both persistent cough due to bronchial involvement (documented by imaging or functional tests) and alterations on high-resolution computed tomography (HRCT) or pulmonary function tests (PFT), possibly, though not necessarily, associated with respiratory symptoms [2]. It is indeed common knowledge that pulmonary involvement in pSS potentially results in small airway abnormalities, interstitial lung disease (pSS-ILD), or lymphoproliferative disorders, often coexisting in patients with pSS [3,4]. These structural abnormalities may cause respiratory symptoms, mainly dyspnea and cough, or they may remain completely silent. Moreover, persistent cough is common in pSS patients because of dryness of upper respiratory airways (xerotrachea), a condition that is excluded from the ESSDAI definition of active pulmonary involvement. Clinical manifestations are, therefore, neither sensitive nor specific to pSS-ILD, and more stringent instrumental and functional criteria are usually employed to define the condition in clinical studies.

The aim of this article is to point out some important aspects of pSS-ILD that are still raising controversies in the available literature, likely as a result of the rarity of the condition combined with the various definitions of ILD applied in the different studies.

## 2. Epidemiology of pSS-ILD

### 2.1. What Is the Real Prevalence of ILD in Patients Diagnosed with pSS?

Respiratory complaints in patients with pSS are common, since various patho-physiological alterations potentially occurring in the disease may represent the underlying cause, as detailed in Section 1. Moreover, attribution of clinical manifestations to the systemic disease is sometimes challenging.

Lung involvement in pSS is not rare, and in older studies, its prevalence ranged widely from 9% to 75%. This was due to high variability in the types of involvement assessed (ILD, small airway disease, or other less frequent abnormalities, such as pleural effusions) and the methods employed to define it (e.g., presence of clinical manifestations, chest X-ray or CT abnormalities, and pathological PFT results). Moreover, classification criteria for enrollment of pSS patients and the time-point considered from the diagnosis of pSS are often different across various studies [3].

In 2015, the EULAR-SS Task Force performed a systematic literature review in order to characterize pSS-related lung involvement. Notably, in unselected pSS patients the overall prevalence of either bronchial or parenchymal lung disease was 16% [5].

Addressing ILD more in detail, in a recent meta-analysis, the pooled prevalence in pSS patients was 13% [6], which is in line with the most commonly reported prevalence of 9–20% [7].

Nevertheless, there is growing awareness that the real prevalence of pSS-ILD is currently underestimated. In most studies, only patients with clinical suspicion of pulmonary involvement undergo HRCT, and asymptomatic patients are usually excluded. Still, older studies performing CT scans in asymptomatic pSS patients showed that lung abnormalities, including ILD features, are detected in up to 65% of subjects [8,9].

Moreover, a conspicuous number of patients develop ILD years before the typical clinical–serological manifestations that lead to the diagnosis of pSS (non-sicca onset pSS) [10,11]. Outside of a rheumatological setting, in the absence of sicca manifestations, the suspicion threshold for pSS is obviously low; therefore, the diagnosis is often missed, or at least delayed, in this subset of patients [12].

### 2.2. What Are the Common Demographic Characteristics of Patients with pSS-ILD?

The ages of pSS-ILD patients in the largest studies range from 55 to 61 years, with a male-to-female ratio of 2:8 [13].

ILD is classically described as a late complication of pSS, with an important study reporting an incidence strongly influenced by disease duration: 10% at 1 year from the diagnosis, 20% after 5 years, and 47% after 15 years [14]. In a meta-analysis, pSS-ILD patients were older than pSS patients without ILD, with a mean difference of 9.25 years in the six studies examined. Moreover, ILD was associated with male gender, with an OR of 1.92 [6]. At the same time, however, there are increasing reports of pSS diagnosis following ILD development by years [15]. These heterogeneous data may suggest the existence of at least two pathogenetically distinct subsets of pSS-ILD patients. The first group represents patients developing ILD as result of a prolonged subclinical local damage to the pulmonary airways and parenchyma in the context of an overt pSS. A second group of subjects may be subjected to immunological dysregulation primarily targeting the lung, even before producing clinically significant alterations in salivary and lachrymal glands leading to pSS diagnosis. Finally, it seems likely that in the near future, increased sensitivity in the diagnosis of pulmonary involvement in pSS patients will lead to a younger age at diagnosis of pSS-ILD.

## 3. Clinical Manifestations of pSS-Related Lung Involvement

### 3.1. When Should Pulmonary Disease Be Suspected in pSS Patients?

Most commonly, symptoms of pSS lung involvement are exertional dyspnea and cough. Less frequently chest pain, sputum production, and fever are reported. Moreover, recurrent infections are common, especially in patients with bronchiectasis [16,17]. These manifestations could actually be related to different types of pulmonary involvement, ranging from dryness of the trachea and main bronchi to small airway disease and the different subtypes of pSS-ILD. These pathologic alterations often coexist in pSS patients and carry very different prognostic and therapeutic implications. As a matter of fact, there are no clinical manifestations specifically pointing at the presence of ILD in pSS patients. The most worrisome consideration, however, is that ILD can occur in pSS patients without causing any respiratory manifestation, at least in early phases. In a Chinese retrospective study, among 66 pSS-ILD patients, only 41 (62.1%) reported respiratory symptoms [18]. Similarly, clinical signs, such as respiratory crackles, may be absent or hard to detect [15].

Notably, the 2021 Consensus Guidelines for Evaluation and Management of Pulmonary Disease in Sjögren’s recommend screening all Sjögren’s patients at baseline with chest X-ray and recommend considering PFT in asymptomatic patients [19]. However, it could be argued that chest X-ray provides very low sensitivity for pSS pulmonary involvement and appears of limited use where pSS-ILD screening is concerned. Bearing these assumptions in mind, we suggest screening all pSS patients (even if asymptomatic) at baseline with PFTs and lung ultrasound, which, in contrast, offer very high sensitivity (see Section 4.2). Whenever pulmonary involvement is clinically suspected, performing an HRCT directly allows one to exclude with certainty pSS-ILD, and it may highlight the presence of additional lymphoproliferative complications, such as amyloidosis or BALT-lymphoma, which would be hardly detected by chest X-ray.

In the future, it would be desirable to identify specific phenotypic subsets of pSS patients at risk for developing ILD, in order to narrow the screening target population, as is already the case for salivary glands MALToma surveillance strategies.

### 3.2. When Should pSS Be Suspected in Patients with Pulmonary Disease?

Sometimes, it can be very challenging to detect the presence of a CTD underlying ILD, mainly because pulmonary involvement is often the dominant organ manifestation and, in some cases, may anticipate other symptoms by years. Furthermore, it is common knowledge that a considerable proportion of patients with apparently idiopathic ILD may present certain features suggesting an underlying systemic autoimmune process, yet not satisfying minimum requirements for the diagnosis of a specific CTD [20]. In 2015, the European Respiratory Society/American Thoracic Society Task Force introduced the definition “interstitial pneumonia with autoimmune features” (IPAF) to describe this subset of patients. Classification criteria for IPAF were proposed, including items suggestive of a CTD (yet not diagnostic) organized into clinical, serologic, and morphologic domains. Notably, sicca symptoms were not included in the clinical domain, as they were considered excessively unspecific [21]. However, a very recent prospective study from Sambataro and colleagues on one of the largest IPAF cohorts showed that pSS was the most common final diagnosis among those patients who progressed towards a specific systemic autoimmune disease during follow-up [22].

Sicca syndrome is actually the main warning symptom of pSS, but other clinical signs may also raise the suspicion of a CTD, including pSS, namely parotid swelling, Raynaud’s phenomenon, and arthritis [23]. Skin inspection may display the presence of pSS cutaneous involvement, including palpable purpura due to cryoglobulinemic vasculitis, annular erythema, or livaedo reticularis [24]. Moreover, routine laboratory analysis may offer important clues—unexplained leukopenia (especially neutropenia), high erythrocyte sedimentation rate, and hypergammaglobulinemia are all common in pSS patients [25].

First-line immunological testing for the screening of ILD patients should always include antinuclear antibodies (ANA), ENA screening, and rheumatoid factor (RF). Furthermore, if the clinical–biohumoral picture is compelling with pSS, a minor salivary gland biopsy (MSGB) may also help diagnosis and prognostic stratification [26]. This is especially true since we recently reported that pSS patients presenting with ILD usually exhibit milder sicca symptoms (probably overshadowed by the respiratory disease) but similar rates of positive anti-Ro and MSGB when compared with classical sicca-onset pSS patients [27].

Finally, thoracic imaging and histologic findings should always be kept in consideration, as some of them are more frequently encountered in CTD than in idiopathic ILD. This is true for the presence of associated signs of bronchial involvement, the presence of lymphoid aggregates, and germinal centers at histology and an LIP pattern, which is suggestive for pSS [21].

In conclusion, another important challenge for the future will be the early identification of pSS patients among those presenting with ILD since it may yield important prognostic and therapeutic implications. In this scenario, the new IPAF cohorts may reveal an important source of pSS-ILD patients, and periodic rheumatologic revaluation of IPAF patients in the contest of a multidisciplinary approach seems advisable.

### 3.3. Do Patients with pSS-ILD Have Distinguishing Clinical or Serologic Features?

Numerous observational studies have tried to identify demographic, clinical, and serologic features potentially associated with the presence of ILD in pSS patients; however, their results are often conflicting. Most studies have failed to highlight clinical features more frequently observed in pSS-ILD patients, aside from signs/symptoms related to the pulmonary ESSDAI domain. Of note, Dong et al. reported a higher prevalence of anti-Ro52 autoantibodies in pSS-ILD patients in a large cross-sectional cohort study. This finding was lately confirmed in a retrospective longitudinal study showing that anti-Ro52 is an independent risk factor for development of ILD [28,29]. In contrast, this association was missed by another observational study analyzing only anti-SSA prevalence, highlighting the importance of assessing separately anti-Ro60 and anti-Ro52 autoantibodies in the evaluation of pSS patients [30,31,32].

In a recent metanalysis conducted on six studies evaluating potential clinical–laboratoristic risk factors for pSS-ILD, the only variables that were found to be statistically associated with ILD were older age, male sex, and elevated CRP [6]. These data are in contrast to the strong association classically reported between other extra-glandular pSS manifestations included in the ESSDAI and immunologic activation markers.

One reason behind these inconsistent results may be the heterogeneity of the cohorts analyzed. In this respect, perhaps it could be useful to stratify the analysis on the basis of the different phenotypes of pulmonary involvement identified on imaging/PFT (or biopsy, if available) [33]. For instance, last year, a retrospective analysis of 15 LIP cases showed higher incidence of anti-Ro60, Ro52, SSB autoantibodies, and hypergammaglobulinemia compared with a larger cohort of pSS-ILD patients [34].

Similarly, we may expect that patients with ILD and marked bronchial involvement will display laboratoristic signs of lymphocytic activation, such as autoantibody positivity or hypergammaglobulinemia. Indeed, histologic lesions in these patients have been shown to be characterized by lymphocytic infiltration and follicular hyperplasia [35]. Of note, a recent study highlighted the association between a high focus score on MSGB and both interstitial and bronchial involvement detected by HRCT [36].

Finally, being expanded from research on IPF, various serum biomarkers and chemokines are emerging as a potential tool for both screening and follow-up of ILD in CTD patients [37]. Until recently, they had been investigated mostly in Scleroderma patients and cohorts with a miscellany of CTD-ILD patients [38,39,40]. Particularly, Krebs von den Lungen (KL-6) is a glycoprotein synthetized by type 2 alveolar cells. KL-6 serum levels are raised in CTD-ILD (including pSS-ILD) patients with respect to non-ILD CTD patients and correlate with the severity of ILD assessed by HRCT and PFT, thus reflecting the extent of the pulmonary damage [41]. In a recent retrospective study on 99 pSS-ILD patients, serum KL-6 even demonstrated prognostic significance, with serum levels > 800 U/mL significantly associated with a worse outcome [42]. However, longitudinal prospective works are required for the validation of this and other biomarkers and for translation in clinical practice. In Table 1, we summarize the findings of some significant works.

## 4. Imaging and Histopathology of ILD in pSS

### 4.1. How Does Radiologic/Histopathologic Pattern Influence the Clinical Picture and Management of pSS-ILD Patients? What Is the Role of Lung Biopsy?

**Figure 1:**
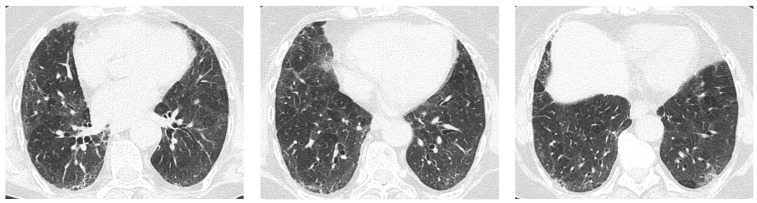
A 68-year-old female pSS patient. Bilateral diffuse ground glass opacities, thickening of subpleural interstitial lobular septa, and traction bronchiectasis compatible with NSIP pattern.

**Figure 2:**
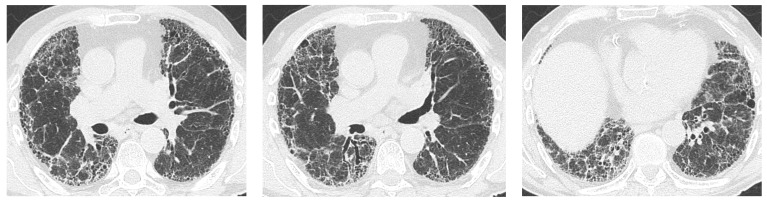
UIP pattern with bilateral subpleural honeycombing and traction bronchiectasis in a 73-year-old male pSS patient.

**Figure 3:**
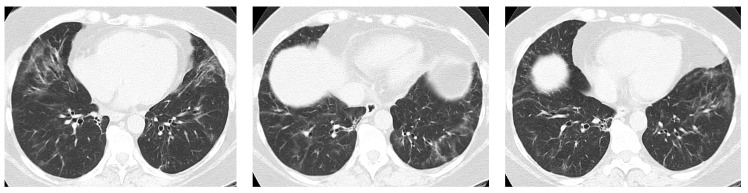
Patchy opacities with peribronchovascular and subpleural distribution in a 64-year-old female pSS patient, suggestive of OP pattern.

**Figure 4:**
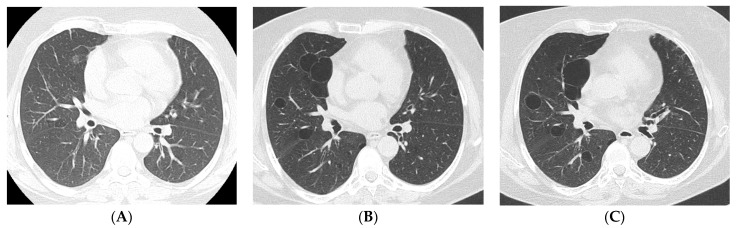
HRCT scan of a 42-year-old female pSS patient in 2008 (**A**) showing only very modest signs of bronchiolitis. She went on to experience recurrent episodes of acute dyspnea and cough, and follow-up HRCT scans in 2011 (**B**) and 2021 (**C**) document progressive formation of thin-walled cysts. The combination of these imaging findings and appropriate clinical context in a pSS patient is virtually pathognomonic of LIP. Of note, confluence of LIP cystic lesions into larger ones (as is shown in (**C**)) exposes the patient to higher risk of complications.

Most studies agree that Non-Specific Interstitial Pneumonia (NSIP) is by far the most common radiologic pattern in pSS-ILD, followed by Usual Interstitial Pneumonia (UIP), Organizing Pneumonia (OP), and Lymphocitic Interstitial Pneumonia (LIP) [10,11]. Figure 1, Figure 2, Figure 3 and Figure 4 show examples of these HRCT patterns in pSS-ILD patients.

In one of the largest populations of pSS-ILD patients, only 60% of patients presented a single HRCT pattern, namely NSIP in 41.7%, UIP in 10.7%, and OP and LIP in 3.9% each. In patients with mixed patterns, the combinations of NSIP with OP, UIP, and LIP patterns were the most common [28]. Notably, fibrotic NSIP is much more common than cellular NSIP in pSS, with reported ratios ranging from 19:1 to 19:3 [45,46].

In a recent systematic review of studies providing histopathological data, NSIP pattern represented 45% of cases, UIP and LIP 16% and 15%, respectively, and OP 7%. Moreover, small airway involvement in the form of bronchiolitis was present in 25% of cases [5].

Studies providing correlations between ILD pattern and clinical–serologic characteristics of pSS patients are rare. UIP pattern was reported to be more frequent in non-sicca onset pSS and is associated with older age and male sex [10,44]. No other clinical features have been associated with specific patterns. Anti-Ro antibodies are more frequently observed in UIP than in NSIP patients; however, a recent study reported higher prevalence of anti-Ro52 in non-UIP than in UIP pSS-ILD patients [44,45,46].

Nevertheless, is it really important to define the morphologic pattern of pSS-ILD patients?

It is well established that in idiopathic ILD, as well as in RA-ILD patients, a UIP pattern carries a higher burden of mortality. In contrast, this is still debated for other CTD-ILD groups, such as in Scleroderma patients [47,48].

In their retrospective analysis of 33 biopsy-proven cases of pSS-ILD, Enomoto et al. found no differences in terms of prognosis between UIP and NSIP patients [45]. Nevertheless, in a recent study of 49 pSS-ILD patients with a follow-up of at least six months, UIP pattern on CT was associated with pulmonary disease progression [44].

Moreover, specific ILD patterns, such as OP and cellular NSIP, are known to portend a good response to steroid and immunosuppressive therapy [49,50,51]. In contrast, immunosuppression has proved to be detrimental in idiopathic UIP (IPF) [52]. Regarding pSS-ILD, Parambil et al. reported on 15 proven biopsy cases treated with steroids and immunosuppressants. In their series, four of four patients with OP, three of five patients with NSIP, and two of three patients with LIP showed functional improvement, while three of three patients with UIP experienced deterioration of lung function [16]. It is important to underline, however, that immunosuppression of IPF patients was associated with increased risk of acute exacerbation and death. On the other hand, we have no evidence that this is also the case for UIP-ILD in pSS. At the present moment, we can only safely conclude that UIP pSS-ILD patients are likely to benefit less from immunosuppressive therapy when compared with pSS-ILD patients exhibiting other patterns.

In clinical practice, HRCT is currently employed as a surrogate, non-invasive instrument to define ILD pattern. In their retrospective series, Ito et al. showed that recognition of NSIP pattern on CT has a high positive predictive value when compared with pathologic analysis. In contrast, non-NSIP patterns on CT were often misdiagnosed. Moreover, the presence of cysts was associated with a pathological diagnosis of MALT-lymphoma and/or amyloidosis [46]. Therefore, while the risk/benefit ratio of surgical lung biopsy is currently considered not favorable in CTD-ILD patients and is not routinely recommended, recently published guidelines for management of pSS-ILD recommend considering biopsy in the event of suspected malignant conditions, amyloid deposition, or infection not responsive to empiric therapy [19].

Accordingly, biopsy may be considered in pSS-ILD patients with suspicious CT patterns. For instance, fixed nodules or consolidations in the context of a cystic pattern, especially in patients with clinical–serological phenotypes at high risk for lymphoma development, would deserve histological characterization.

A much safer alternative to surgical lung biopsy is traditional transbronchial biopsy (TBB). However, it is known that TBB has a very low diagnostic yield for IPF, and it has been shown to correlate very poorly with surgical lung biopsy in a small series of pSS-ILD patients. Particularly, small airway disease was entirely missed by traditional TBB [35,53]. More recently, transbronchial criobiopsy (TBCB) has emerged as a good compromise, with a meta-analysis proving a diagnostic yield for the suspicion of ILD of 0.81 and much lower rates of mortality and procedural complications compared with surgical biopsy [54].

Notably, in clinical practice, immunosuppressive therapy for CTD-ILD is especially considered for HRCT patterns where there is evidence of active inflammation. However, this approach is borrowed mainly from the experience matured in idiopathic ILD treatment, and prospective studies supporting it are lacking. Considering the implementation of the therapeutic armamentarium with the new antifibrotic drugs, TBCB may radically change the approach to the management of CTD-ILD. Examination of TBCB samples might help to distinguish ILD cases with inflammatory infiltrates that may benefit from immunosuppression, mainly from fibrosing conditions.

Moreover, availability of new techniques of tissue transcriptomic analysis may improve our knowledge of pathogenetic pathways and allow identification of new potential targets for tailored therapies [55].

### 4.2. Does Lung Ultrasound (LUS) Have a Defined Role in pSS-ILD Screening and Follow-Up?

LUS is a low cost and non-invasive technique, originally developed and employed in intensive care and cardiologic settings. LUS allows the dynamic study of lung parenchyma through the assessment of ultrasound artifacts [56,57].

B lines are defined as hyperechoic, ray-like, vertical ring down artifacts, arising from the pleural line, obliterating other background LUS artifacts and moving with lung sliding. They are produced by the great acoustic mismatch between air in the alveoli and interstitial septa filled by fluid (in case of interstitial edema) or inflammatory/fibrotic infiltrates. Therefore, B lines (previously named Comet Tail Artifacts by Lichtenstein et al.) are regarded as a markers of “interstitial syndrome”. Up to three B lines for scan may be found in healthy subjects, and their number increases with age and in dependent lung zones [58].

Clinical findings, distribution of B lines, and their possible association with the other cardinal findings suggestive of pulmonary fibrosis, namely pleural irregularities (PI), allow for the differentiation of cardiogenic interstitial edema from fibrotic ILD.

In 2003, Angelika Reißig and Claus Kroegel first described the application of LUS to the study of interstitial lung changes in ILD patients (mainly IPF). Since then, LUS has progressively emerged as a new tool for the assessment of lung pathology in CTD patients [59,60]. Most reports have focused on Systemic Sclerosis (SSc) patients, demonstrating the positive correlation of LUS B lines with fibrosis on HRCT scored with the Warrick method, and the negative correlation with Total Lung Capacity (TLC) and DLCO on PFT [61,62].

Moreover, analyzing a cohort of SSc with a large proportion of VEDOSS, Barskova et al. showed that LUS allows early detection of ILD, even in patients with disease onset, showing a negative predictive value of 100% compared with HRCT [63].

Other studies extended these findings to miscellaneous CTD cohorts, including pSS-ILD patients [64,65]. Guisado-Vasco et al. showed that LUS exhibited 100% sensitivity in detecting ILD in a small SS population with either respiratory symptoms or PFT alterations [66].

Similarly, our group reported that pleural irregularities assessed by LUS potentially identify ILD even in a subclinical phase, in asymptomatic pSS patients [67].

**Figure 5:**
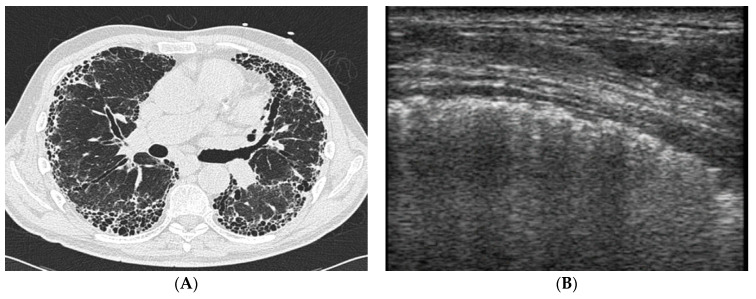
(**A**) HRCT scan of a patient with pSS showing bilateral subpleural honeycombing and traction bronchiectasis compatible with UIP pattern. (**B**) LUS performed on the same patient, with a 5–12 MHz linear probe showing marked irregularities of the pleural line.

In conclusion, LUS is definitely a very sensitive, low-cost, non-invasive, ionization-sparing technique for the early screening of CTD patients at risk for lung involvement [68]. While recent guidelines suggest performing basal chest X-ray and PFT at pSS diagnosis, HRCT of the lung is recommended only in the setting of overt clinical manifestations [19]. Given its high sensitivity and negative predictive value, LUS may be performed as a screening test to identify patients deserving HRCT assessment; however, further validation studies are needed before LUS is implemented in routine clinical practice. Figure 5 shows examples of LUS assessment in pSS patients.

## 5. Prognosis and Treatment of Progressive pSS-ILD

### 5.1. What Is the Prognosis of Patients with pSS-ILD? How Can We Identify Progressive ILD Phenotypes Deserving Pharmacologic Treatment?

Although lung involvement in pSS patients is typically considered mild in comparison with other CTDs, most studies suggest a reduced survival in pSS-ILD patients [16,32,69].

Recently, in a retrospective study conducted on a population of 178 hospitalized patients with pSS-ILD, Gao et al. reported a 10-year survival rate of 81.7%, with respiratory failure being the cause of death in 67% of cases [70]. Moreover, ILD carries a significant morbidity burden, with a negative impact on quality of life for pSS patients [71].

The clinical course of pSS-ILD has been described mostly in retrospective studies, showing improvement or stabilization of the pulmonary picture in considerably more than 50% of conventionally treated patients [16,30,72].

Regarding the management of pSS-ILD patients, as for other CTD-ILD patients, clinical presentation is clearly the most important variable to be assessed. Indeed, 2019 EULAR recommendations suggest considering systemic pharmacologic therapy for patients with moderate/severe ESSDAI in the lung domain, i.e., symptomatic patients (at least NYHA II dyspnea) or with significant impairment in PFT (FVC < 80% or DLCO < 70%) [73]. This line was lately confirmed by the more recent Consensus Guidelines for Evaluation and Management of Pulmonary Disease in Sjögren’s, which recommend that asymptomatic patients with substantially normal lung function and imaging should undergo close follow-up of PFTs in order to detect a clinically significant decline in lung function deserving pharmacologic intervention [19].

Considering the well-known side-effects and costs of immunosuppressive and anti-fibrotic therapy, anticipating the trajectory of the pulmonary function of pSS-ILD is crucial for the management of these patients. In this respect, some clues may come from recent studies investigating baseline risk factors for ILD progression and outcome, which we summarize in Table 2. Specifically, baseline pulmonary function expressed by forced vital capacity (FVC) and arterial blood gas results, as well as extension of pulmonary involvement on HRCT, invariably predict ILD progression and mortality across different studies. In contrast, presence of a UIP pattern on HRCT was associated with a worse prognosis in some studies [16,44] but not in others [45,70]. Notably, however, these parameters are indicative of a poor prognosis, while they do not predict a good response to therapy—patients included in the aforementioned studies were indeed treated according to standard of care. Therefore, the next step for the optimal manage of pSS-ILD patients should be the identification of biohumoral or imaging markers, reflecting active inflammation potentially targeted by anti-inflammatory therapy and/or ongoing lung fibrosis that would benefit from anti-fibrotic therapy. In this regard, a novel nuclear imaging approach based on radio-labeled fibroblast activation protein inhibitor seems very promising for the evaluation of ILD activity [74].

### 5.2. What Are the Most Commonly Employed Treatment Approaches for pSS-ILD? What Therapeutic Innovations Can We Expect Based on New Insights into pSS Pathogenesis?

When it comes to pharmacological treatment, RCT and comparative studies on pSS-ILD patients are still lacking. Therefore, the best evidence available comes from small case series, and management strategies are also based on RCTs conducted on SSc and IPF patients. Most pSS-ILD patients are at least initially treated with steroids at a dosage of 0.5 mg/kg/day of Prednisone (treatment with 1 mg/kg/day is reported in an older series from Parambil et al.), while steroid pulses are not usually employed. Several immunomodulant and immunosuppressive agents have been administered as steroid-sparing agents, most commonly including Hydroxychloroquine, Azathioprine (AZA), oral or IV Cyclophosphamide (CFX), Mycophenolate Mofetil (MMF), and Rituximab (RTX) [16,30,35,42,45,46,72]. Moreover, use of the anti-fibrotic agent Pirfenidone in pSS-ILD with UIP pattern was reported in 2017 [75]. More recently, He et al. administered Pirfenidone to 7 of the 837 pSS-ILD patients of their cohort [72]. Of note, the INBUILD trial compared another anti-fibrotic agent, Nintedanib, with placebo in patients with non-IPF ILD with a progressively fibrosing phenotype, including CTD-ILD patients. Nintedanib-treated patients presented a lower decline rate in FVC, with a more marked difference observed in patients with a UIP-like fibrosing pattern [76].

Both 2020 EULAR recommendations for the management of pSS [73] and 2021 Consensus Guidelines for Evaluation and Management of Pulmonary Disease in Sjögren’s [19] recommend pharmacological treatment for pSS-ILD patients with moderate/severe lung involvement according to the ESSDAI. The choice of the proper pharmacologic approach should consider several variables, including comorbidities and concomitant organ involvement; however, first and foremost, the specific phenotype of pulmonary involvement should be considered. Of note, EULAR recommendations specify that steroid therapy is especially indicated in the more inflammatory ILD patterns (LIP and OP), while NSIP—and even more so, UIP patterns—are less likely to benefit from steroids [73]. MMF and AZA are currently regarded as the first-line steroid-sparing agents. In patients with insufficient response to first-line therapy, ILD phenotype and pattern should be reassessed. In the case of predominant inflammation, second-line immunosuppressive therapy with RTX or calcineurin inhibitors or CFX is advised, while antifibrotic agents are recommended mainly for progressive fibrosing ILD [19].

Pathogenetic mechanisms related to pSS-ILD are likely to resemble, at least partially, those leading to lymphocytic infiltration and eventual architectural disruption and fibrotic degeneration of the salivary glands of pSS patients [17].

For instance, the IL17/IL22-TGFbeta1 pathway is thought to mediate epithelial–mesenchimal-transition-dependent fibrosis both in chronic fibrotic lung diseases (including CTD-ILD and IPF) and in the salivary gland tissue of pSS patients, who, moreover, can display elevated serum levels of IL17 and IL22 [77,78,79]. Interestingly, inhibition of IL-17 led to resolution of pulmonary fibrosis in both in vitro and in vivo models [80]. Components of this axis (including signal transducers) may, therefore, represent promising therapeutic targets [81,82].

Moreover, Ectopic Lymphoid Structures (ELS), a hallmark of pSS lymphocytic sialadenitis, are also observed in pulmonary biopsy specimens of pSS-ILD cases, likely exerting a pathogenetic role [83]. T-B lymphocyte cooperation is essential for ELS development and represents the target of Iscalimab, an anti-CD40 monoclonal antibody, that has proved to significantly reduce ESSDAI score in a preliminary phase IIa RCT and may represent a revolutionary innovation in pSS treatment [84].

Finally, type 1 IFN plays a pivotal role in the early phase of pSS autoimmune epithelitis, and pSS patients often exhibit an IFN-signature [85]. Type 1 IFN signaling is mediated by the JAK-STAT pathway, whose modulation with the novel JAK-inhibitors has proved efficacious in different autoimmune diseases. Growing evidence indicates that JAK-STAT signaling is also involved in both pro-inflammatory and pro-fibrotic processes in ILD, and JAK-inhibitors are being successfully employed for the treatment of both COVID-19 disease and rapidly-progressive CTD-ILD [86,87,88,89,90]. Recently, a phase II RCT of Filgotinib (a Jak1-inhibitor) in pSS failed to reach its primary endpoint, consisting of a composite outcome measure; however, a sub-analysis of the trial showed a trend toward clinical efficacy in patients with severe disease [91]. Jak-I’s potential role in pSS-ILD management may deserve to be further evaluated by future studies.

### 5.3. Is Lung Transplantation a Feasible Option for pSS-ILD Patients with Advanced Lung Disease?

Progressive fibrosing ILD in the context of pSS uncommonly leads to severe chronic respiratory failure. Chronic autoimmune diseases have often been considered a relative contraindication to lung transplantation. Possible concerns pertain mostly to the theoretic increased risk for transplant rejection due to immune system hyperactivation [92]. Moreover, autoimmune patients are often exposed for a long period to steroids and immunosuppressive treatment, which may affect the outcome of the organ transplant.

However, interestingly, various studies reported similar outcomes between IPF- and CTD-related ILD patients undergoing pulmonary transplantation because of end-stage lung disease [93,94,95]. Similarly, a large retrospective cohort study analyzed the outcome of non-SSc CTD patients with ILD, including pSS-ILD, who underwent lung transplantation. Compared with control IPF subjects, CTD-ILD patients exhibited similar survival and risk of organ rejection [96]. Furthermore, there are increasing reports regarding pSS-ILD patients who successfully received lung transplant with encouraging results [97].

Ideally, progressive fibrosing pSS-ILD patients should be managed with early immunosuppressive and/or antifibrotic treatment in order to prevent end-stage disease. However, on the basis of available data, lung transplantation should be considered as a feasible option for pSS-ILD patients with advanced disease.

## 6. Conclusions

In this paper, we discussed some hot topics regarding lung involvement and specifically ILD in the context of pSS, underlying aspects that, in our view, deserve to be addressed by future research work. ILD is a common, and likely underestimated, clinical manifestation of pSS.

Diagnosis of pSS-ILD is not always straightforward due to a great proportion of mildly symptomatic patients, current lack of specific biomarkers, and a physiological selection bias that leads patients with severe ILD and mild sicca symptoms to respiratory units, which are not always equipped with a structured collaboration with trained rheumatologists. Noteworthy, recognition of pSS in ILD patients is clinically relevant since physicians should be aware of the risk of other potential visceral involvement and hematological malignancy. The correct classification of these patients is also crucial for research purposes in order to further improve current knowledge regarding clinical presentation, potential biomarkers, and appropriate management of pSS-ILD.

## Figures and Tables

**Table 1:** Studies investigating ILD association with specific characteristics of pSS patients.

Authors, Year	No. of ILD Patients	Features Associated with ILD Presence in pSS		
		Demographic	Clinical	Laboratoristic
Shi et al., 2020 [43]	168	-	Higher ESSDAI	Ca 15.3 and CEA levels
Zhang et al., 2020 [44]	85	Older age, disease duration	Fever, xerophtalmia, xerostomia	WBC, CRP, and IgG levels
Buvry et al., 2020 [29]	31	-	-	Anti-Ro52
Gao et al., 2018 [32]	165	Older age	-	RF and CRP
Dong et al., 2018 [28]	206	Older age, smoking	Dry cough, clubbing, weight loss	Anti-Ro52, higher LDH and ESR levels, and lower albumin/globulin ratio
Wang et al., 2018 [31]	158	Male sex, older age, smoking	-	ANA
Roca et al., 2017 [30]	21	Older age	Raynaud’s phenomenon, esophageal involvement	-
Li et al., 2015 [18]	66	-	-	Anti-SSA and low C3 levels

ESSDAI—EULAR Sjögren’s syndrome disease activity index; Ca 15.3—Cancer antigen 15.3; CEA—Carcinoembryonic antigen; WBC—White blood count; CRP—C reactive protein; IgG—Immunoglobulin G; RF—Rheumatoid factor; LDH—Lactate dehydrogenase; ESR—Erythrocyte sedimentation rate; ANA—Antinuclear antibodies; C3—Complement C3 levels.

**Table 2:** Studies investigating risk factors for progression and prognosis of pSS-ILD.

Studies	Risk Factors	Outcome Assessed	Prognosis
He et al., 2021 [72]	LDH, non-sicca onset, low FVC *	ILD progression *	Progression of ILD in 38.6% of cases
Gao et al., 2021 [70]	TLCO/VA, MEF25, PaO_2_	Mortality	10-year survival rate of 81.7%
Zhang et al., 2020 [44]	ESR, UIP pattern	ILD progression at 6 months †	Progression of ILD in 20.4% of cases
Kamiya et al., 2019 [42]	Age, KL-6, low FVC	Mortality	5-year survival rate of 89.8% 10-year survival rate of 79%
Enomoto et al., 2013 [45]	PaCO_2_, extension of reticulations on HRCT, severity of fibroblastic foci	Mortality	5-year survival rate of 87.3%
Ito et al., 2005 [46]	PaO_2_, absence of microscopic honeycombing	Survival at 5 years	5-year survival rate of 84%

* Assessed by combination of PFT, HRCT, and pulmonary symptoms; † assessed by PFT. FVC—Forced vital capacity; TLCO/VA—Transfer factor of carbon monoxide/alveolar volume; MEF25—Maximum expiratory flow at 25%.

## Data Availability

Not applicable.
